# Evidential Decision Tree Based on Belief Entropy

**DOI:** 10.3390/e21090897

**Published:** 2019-09-16

**Authors:** Mujin Li, Honghui Xu, Yong Deng

**Affiliations:** School of Computer Science and Engineering, University of Electronic Science and Technology of China, Chengdu 610054, China

**Keywords:** decision, belief entropy, Deng entropy, basic belief assignment, fuzzy data classification

## Abstract

Decision Tree is widely applied in many areas, such as classification and recognition. Traditional information entropy and Pearson’s correlation coefficient are often applied as measures of splitting rules to find the best splitting attribute. However, these methods can not handle uncertainty, since the relation between attributes and the degree of disorder of attributes can not be measured by them. Motivated by the idea of Deng Entropy, it can measure the uncertain degree of Basic Belief Assignment (BBA) in terms of uncertain problems. In this paper, Deng entropy is used as a measure of splitting rules to construct an evidential decision tree for fuzzy dataset classification. Compared to traditional combination rules used for combination of BBAs, the evidential decision tree can be applied to classification directly, which efficiently reduces the complexity of the algorithm. In addition, the experiments are conducted on iris dataset to build an evidential decision tree that achieves the goal of more accurate classification.

## 1. Introduction

Decision trees are one of the efficient techniques that are widely used in various areas, like machine learning, image processing, and pattern recognition. Decision trees are good due to having better comprehensibility of classification in terms of extracting from feature-based samples [1,2,3]. In addition, decision trees were not only proven efficient in many fields [4], but also have less parameters [5]. There are two main rules considered in the process of building decision trees [6]. One is the stopping criterion to determine when to stop the growth of tree and generate leaf nodes [7]. The other is how to assign class labels in leaf nodes [8]. The first rule means that the growth of the tree should be ended [9] if all samples belongs to the same class [9]. The second rule emphasizes the importance of setting a threshold [10]. There exist many methods of decision trees, such as ID3 [7], C4.5 [11,12], and CART [10].

However, all rules in the processing of decision trees are under certain situations; while the real world is filled with uncertainty [13,14,15,16]. Thus, when it deals with uncertain issues, all the rules should take uncertainty into consideration. Dempster-Shafer evidence theory (D–S) [17,18] is widely used in many applications such as decision making [19,20,21,22,23,24], evidential reasoning [25,26,27,28], information fusion [29,30], pattern recognition [31,32,33], fault diagnosis [34,35,36,37], risk evaluation [38,39,40], network analysis [41], conflicting management [42,43,44,45], uncertainty modeling [46,47,48,49,50], and so on [51,52,53,54]. In the D–S evidence theory, Basic Belief Assignment (BBA) measures the uncertainty. Deng entropy [55] is proposed to quantify the uncertain measure of BBA.

Some works combined with evidence theory and decision trees are presented [56,57,58,59,60], but, motivated by the idea of building decision tree based on Pearson correlation coefficient [61] and the proposed Deng entropy instead of information entropy [62,63,64,65,66], in this paper, the evidential decision tree is proposed for classification of fuzzy data sets using BBAs, which are applied directly for classification instead of using combination rules for classification indirectly. That is to say, the evidential decision tree is constructed for classification directly based on BBAs rather than using combination rules, which not only reduce the complexity of algorithm but also avoid designing the combination rules, which is always complicated. Moreover, the proposed evidential decision trees are much more efficient than traditional decision tree methods, illustrated by the analysis of experiments with the iris data set and wine data set.

The organization of this paper is introduced briefly as follows. Section 2 presents the introduction of preliminaries. The building of the evidential decision tree is shown in Section 3. Experiments are conducted in Section 4. This paper ends with the conclusion in Section 5.

## 2. Preliminaries

In this section, D–S evidence [17,18], Deng Entropy [55], and Pearson’s correlation coefficient based on the decision tree (PCC-Tree) [61] are briefly introduced. D–S evidence theory is introduced to present the definitions in terms of uncertain problems. Additionally, the Deng entropy is introduced to calculate the uncertain degree of BBAs. Finally, PPC-Tree is followed by the proposed method, replacing Pearson’s correlation coefficient with Deng entropy to build an evidential decision tree.

### 2.1. D–S Evidence Theory

Handling uncertainty is an open issue, and many methods have been developed [67,68,69]. In D–S evidence theory [17,18], $\Theta =(A_{1},A_{2},A_{3},\cdots ,A_{n})$ is a frame of discernment. $A_{i}\left(1\leq i\leq n\right)$ represents the identification of every element in the framework.

Basic Belief Assignment (BBA), a mass function, is one of the most important definitions of D–S evidence theory and many operations are presented based on it such as negation [70,71], divergence measure [72], and correlation [73]. BBA has two features: m(∅)=0 and $\sum_{A\subseteq \Theta }m\left(A\right)=1$. It should be mentioned that the BBA of an empty set in classical evidence theory is zero [74].

For the same evidence, different Basic Belief Assignments will be obtained due to different independent evidence sources. Assuming the frame of discernment is Θ, $m_{1},m_{2},m_{3},\cdots m_{n}$ are *n* different BBAs which are all independent. According to Dempster’s combination rule, the result is presented as follows:

(1) $$\begin{array}{c} m=m_{1}⨁m_{2}⨁m_{3}⨁\cdots ⨁m_{n}, \end{array}$$

(2) $$m\left(A\right)=\left\{\begin{array}{cc} 0 & \;if\;A=\emptyset ;\\ K^{-1}\sum_{\cap A_{j}=A}\prod_{i=1}^{n}m_{i}\left(A_{j}\right) & \;otherwise, \end{array}\right.$$

*K* is normalization factor, which is defined as follows:

(3) $$K=1-\sum_{⋂A_{j}=\emptyset }\prod_{i=1}^{n}m_{i}\left(A_{j}\right).$$

The reliability factor α(α∈[0,1]) is given to construct the discounted mass function ${}^{\alpha }m$, *m* is one of the BBAs on the identification frame Θ:

(4) $$\begin{array}{c} {}^{\alpha }m\left(A\right)=\left\{\begin{array}{cc} \alpha m(A) & if\;A\subset \Theta ,A\neq \Theta ;\\ 1-\alpha +\alpha m(\Theta ) & otherwise, \end{array}\right. \end{array}$$

### 2.2. Deng Entropy

Inspired by Shannon Entropy, a new uncertainty method called Deng Entropy is proposed [55]:

(5) $$E_{d}\left(m\right)=-\sum_{A\subseteq \Theta }m\left(A\right)\log_{2}\frac{m(A)}{2^{\left|A\right|}-1}.$$

As shown in the above definition, different from the classical Shannon entropy, the belief for each focal element *A* is divided by $\left(2^{\left|A\right|}-1\right)$, which means the potential number of states in *A*. Through a simple transformation, it is found that Deng entropy is actually a type of composite measure, as follows: If the quotient rule of logarithm transformation of Deng Entropy is carried out, it is actually a comprehensive measurement:

(6) $$E_{d}\left(m\right)=\sum_{A\subseteq \Theta }m\left(A\right)\log_{2}\left(2^{\left|A\right|}-1\right)-\sum_{A\subseteq \Theta }m\left(A\right)\log_{2}m\left(A\right),$$

where the first term could be explained as a measure of total nonspecificity in the mass function *m*, and the second term could be interpreted as the measure of discord of the mass function among distinct focal elements.

### 2.3. PCC-Tree

During building decision trees, the Pearson’s correlation coefficient can be used as the optimal splitting point—PCC-Tree [61].

Following the idea of building the traditional decision tree, one new type of decision tree was reconstructed by Pearson’s correlation coefficient through a top-down recursive way. The detailed constructing process can be found in Algorithm 1.

**Algorithm 1** Constructing a PCC-Tree**Require:** A root node $X={\left\{x_{i}\right\}}_{i=1}^{N}$, where $x_{i}$ is the i th instance with *n* condition attributes ${\left\{A_{k}\right\}}_{k=1}^{n}$ and one decision attribute D; the stopping criterion ε.**Ensure:** A PCC-Tree. **if** the samples in *X* belong to some class **then**  Mark *X* as a leaf node and assign the class as its label.  **return**. **end if** **for** each attribute $A_{k},k=1,2,\cdots ,n$ in *X* **do**  **for** each value $c_{i}$ in $A_{k}$ **do**   Compute the Pearson’s correlation coefficient *P* of two vectors: $P_{c_{j}}\left(A_{k}\right)=P(V\left(A_{k},c_{j}\right)$ and V(D)),   where *P* denotes Pearson’s correlation coefficient and *V* denotes one vector.  **end for**  $c_{jk}^{*}=\arg\max_{c_{j}}P_{c_{j}}\left(A_{k}\right)$. **end for** Get the best attribute $A_{k^{*}}$ and the splitting point $c_{k^{*}}$, where $k^{*}=\arg\max_{k}P_{c_{j}}\left(A_{k}\right)$. Suppose p(X) is the proportion of samples covered by *X*. **if** p(X)<ε **then**  Mark *X* as leaf node.  Assign the maximum class of samples in X to this leaf node.  **return** **else**  Split *X* into two subsets $X_{1}$ and $X_{2}$, based on $A_{k^{*}}$ and $c_{k^{*}}$.  **if** $p(X_{1})==0$ or $p(X_{2})==0$ **then**   Mark *X* as a leaf node.   Assign the maximum class of samples in *X* to this leaf node.   **return**  **end if**  Recursively search the new tree nodes from $X_{1}$ and $X_{2}$ by Algorithm 1, respectively. **end if**


## 3. Proposed Method

Evidential decision tree is introduced in this section. Motivated by the idea of building a decision tree based on Pearson’s correlation coefficient, the Deng Entropy is calculated as a measure in splitting rules processing the decision tree. The difference is that the relation between the probability distributions of attributes and the probability distribution of decision attributes can be measured by Pearson’s correlation coefficient, but BBAs can not in terms of uncertainty. Thus, the Deng Entropy is proposed in this paper, as a measure of splitting rules processing in the decision tree. In the end, the decision tree is built in the situation of uncertainty.

### 3.1. BBA Determination

It is an open issue to determine the BBAs of attributes. In this paper, one of them is chosen to determine the BBAs [75]. The procedures are introduced in detail as follows.

1. **Step 1:** Normality test is carried out for each attribute column from each training set class. Consider a case where there are *N* samples in each class i(i=1,2,⋯,n) in the training set, and the attribute j(j=1,2,⋯,k) column (length *N*) are normality tested to get a Normality Index for the attribute *j* of class *i*, donated as $NI_{ij}$ (binary expression). If $NI_{ij}=0$, it means the selected attribute obeys the experimental assumption. Otherwise, if $NI_{ij}=1$, it represents that the attribute does not follow normal distribution. Transformation of the original data to an equivalent normal space will occur when condition $\sum_{i=1}^{n}NI_{ij}\geq \frac{n}{2}$ is adopted.
2. **Step 2:** Calculate the value of the mean and the sample standard deviation of each sample for selected class and selected attribute.
  (7) $$\begin{array}{cc} \mu_{ij} & =\frac{1}{N}\sum_{l=1}^{N}x_{ijl},\\ s_{ij} & =\sqrt{\frac{1}{N-1}\sum_{l=1}^{N}{\left(x_{ijl}-\mu_{ij}\right)}^{2}}. \end{array}$$
  $x_{ijl}$ is the sample value of the $j_{th}$ attribute from the $l_{th}$ sample in class *i*. Thus, obtain the corresponding normal distribution function:
  (8) $$f\left(x;\mu_{ij},s_{ij}^{2}\right)=\frac{1}{\sqrt{2\pi s_{ij}^{2}}}e^{-{\left(x-\mu_{ij}\right)}^{2}/\left(2s_{ij}^{2}\right)}.$$
  For each attribute, *n* normal distribution functions (or curves) can be obtained as models of different classes in the specific attribute.
3. **Step 3:** Determine the relationship between the test sample and the normal distribution models. Choose a sample from the test set, the *n* intersection of the selected sample is obtained by calculating the intersection of $x_{j}\left(j=1,2,\cdots ,k\right)$ and the *n* normal distribution functions $f(x;\mu_{ij},s_{ij}^{2})$.
4. **Step 4:** For the *n* intersections of the selected attribute j(j=1,2,⋯,k), $y_{r}\left(r=1,2,\cdots ,n\right)$ ranks them in decreasing order, $w_{r}\left(r=1,2,\cdots ,n\right)$. For $w_{r}\left(r=1,2,\cdots ,n\right)$, its corresponding class (i.e., the class the normal distribution curve belonged to) can be denoted as $C_{r}\left(r=1,2,\cdots ,n\right)$. Assign $w_{r}\left(r=1,2,\cdots ,n\right)$ to a proposition by the following rule:
  (9) $$\begin{array}{c} m\left(\left\{C_{1}\right\}\right)=w_{1},\\ m\left(\left\{C_{1},C_{2}\right\}\right)=w_{2},\\ \cdots \\ m\left(\left\{C_{1},C_{2},\ldots ,C_{n}\right\}\right)=m\left(\Theta \right)=w_{n}. \end{array}$$
  If $w_{r}=w_{r+1}=\cdot \cdot =w_{q}$, then $m\left(\left\{C_{1},C_{2},\ldots ,C_{q}\right\}\right)=\sum_{p=r}^{q}w_{p}$. If $x_{j}$ is a missing value, its corresponding BBA will be assigned as m(Θ)=1, which means that the attribute is regarded as ignorance.

### 3.2. Deng Entropy Calculation

In this part, Deng Entropy is used to measure the degree of uncertainty of BBAs in each attribute. Deng Entropy will then be used as the measure of splitting rules. According to Equations (5) and (9), the Deng Entropy can be calculated as follows:

(10) $$E_{d}\left(m_{j}\right)=-\sum_{w\subseteq \Theta }m_{j}\left(w\right)\log_{2}\frac{m_{j}\left(w\right)}{2^{\left|w\right|}-1}.$$

### 3.3. Evidential Decision Tree Construction

Based on the above equations, the decision tree based on Deng Entropy can be constructed in a top-down recursive way, which follows the traditional progress of decision trees. Firstly, the algorithm is proposed to find the best attribute for splitting rules shown in Algorithm 2.

**Algorithm 2** Splitting Rules based on Deng Entropy**Require:** A root node $X={\left\{x_{i}\right\}}_{i=1}^{N}$, where $x_{i}$ is the ith instance with *n* condition attributes ${\left\{A_{k}\right\}}_{k=1}^{n}$ and one decision attribute D; the stopping criterion: Until all conditional attributes are used up.**Ensure:** An Evidential Decision Tree. **if** the samples in *X* belong to some class **then**  Mark *X* as a leaf node and assign the class as its label.  **return**. **end if** **for** each attribute $A_{k},k=1,2,\cdots ,n$ in *X* **do**  Computer Deng Enropy $E_{d}\left(A_{k}\right)$ according to Equation (10)  $E_{d}\left(A_{k}\right)=-\frac{\sum_{i=1}^{N}\sum_{w\subseteq \Theta }c_{i}^{k}\left(w\right)\log_{2}\frac{c_{i}^{k}\left(w\right)}{2^{\left|w\right|}-1}}{N}$$c_{i}^{k}$ represent a BBA for the instance *i* of attribute $A_{k}$  $E_{d}\left(A_{k^{*}}\right)=argmin_{A_{k}}E_{d}\left(A_{k}\right)$. The smaller the entropy value, the better the subsequent division. **end for** Get the best attribute $A_{k^{*}}$ and the splitting point $c^{k^{*}}$.

Secondly, Algorithm 3 is proposed to classify samples by maximum value and minimum value of training set and find the child nodes of decision tree. In this section, the implementation of the algorithm is illustrated by taking the case of only three classes as an example. Similar to Algorithm 3, branches only need to be added when the number of classes increases.

**Algorithm 3** Construct an Evidential Decision Tree**Require:** Set attributes as Features. Set classes as A,B,C, etc.**Ensure:** An Evidential Decision Tree. **for** All samples **do**  **for** All Feature **do**   **if** Feature≥max($B_{max},C_{max},A_{min},\cdots$)   && Feature1≤min($A_{max},B_{min},C_{min},\cdots$) **then**    **return** A.   **end if**   **if** Feature≥max($A_{max},C_{max},B_{min},\cdots$) && Feature1≤min($B_{max},A_{min},C_{min},\cdots$) **then**    **return** B.   **end if**   **if** Feature≥max($A_{max},B_{max},C_{min},\cdots$) && Feature1≤min($C_{max},A_{min},B_{min},\cdots$) **then**    **return** C.   **end if**  **end for** **end for**

#### An Illustration For Evidential Decision Tree Construction

Assuming that there is a set of training instance $S=\{e_{1},e_{2},\cdots ,e_{N}\}$, $\lambda =\{A_{1},A_{2},\cdots ,A_{n}\}$ is a set of evidential test attributes, and each attribute $A_{k}$ is represented by a belief function on the set of possible terms. Let D be the decision attribute and the members of it compose the frame of discernment Θ.

In order to better illustrate the implementation of the algorithm in the process of building a decision tree based on Deng entropy, a numerical example shown in Table 1 is given to illustrate the meaning of each notations.

In this example, there are two test attributes and one decision attribute. According to proposed approach steps, the Deng entropy should be calculated under these circumstances.

In the implementation of Algorithm 2, $A_{k}$ means each attribute, $c_{i}^{k}$ represents the value of each focal element in the identification framework for the instance $e_{i}$ of attribute $A_{k}$. In other words, $c_{i}^{k}$ is the term $m_{j}\left(w\right)$ in Equation (10).

For the two properties of Table 1, there are some specific notation representations:

- D=Θ={A,B,C};
- $A_{1}=\left\{Weather\right\},A_{2}=\left\{Humidity\right\}$;
- ${c_{1}}^{1}=\left\{\left\{A\right\}:0.8,\left\{A,B\right\}:0.2,\left\{A,B,C\right\}:0\right\}\;{c_{3}}^{2}=\left\{\left\{A\right\}:0.4,\left\{A,C\right\}:0,\left\{A,B,C\right\}:0.6\right\}$;
- $E\left(A_{1}\right)=-\frac{\sum_{w\subseteq \Theta }c_{1}^{1}\left(w\right)\log_{2}\frac{c_{1}^{1}\left(w\right)}{2^{\left|w\right|}-1}+\sum_{w\subseteq \Theta }c_{2}^{1}\left(w\right)\log_{2}\frac{c_{2}^{1}\left(w\right)}{2^{\left|w\right|}-1}+\cdots +\sum_{w\subseteq \Theta }c_{N}^{1}\left(w\right)\log_{2}\frac{c_{N}^{1}\left(w\right)}{2^{\left|w\right|}-1}}{N}$.

By comparing the calculation result of each attribute of Deng entropy, Algorithm 2 can find the father nodes of the decision tree, and Algorithm 3 is used to find the child of the decision tree.

## 4. Experiment

### 4.1. The Application of the Proposed Method

The Iris data set contains three classes, and each classes has 50 samples. These BBAs are used to generate the evidential decision tree instead of being combined to do classification work.

First, the iris data set is used to generate the BBAs shown in Table 2. Set classes as A,B,C, etc.

Second, samples should be classified simply by maximum value and minimum value of iris dataset used in Algorithm 3, shown in Table 3. Classifications of wines are shown in Table 4.

Then, according to the BBAs in Table 2, the Deng entropy is calculated, as shown in Table 5, which will be used as the measure of splitting rules to find the best splitting attribute. Deng entropy for wines are shown in Table 6.

**Figure 1:**
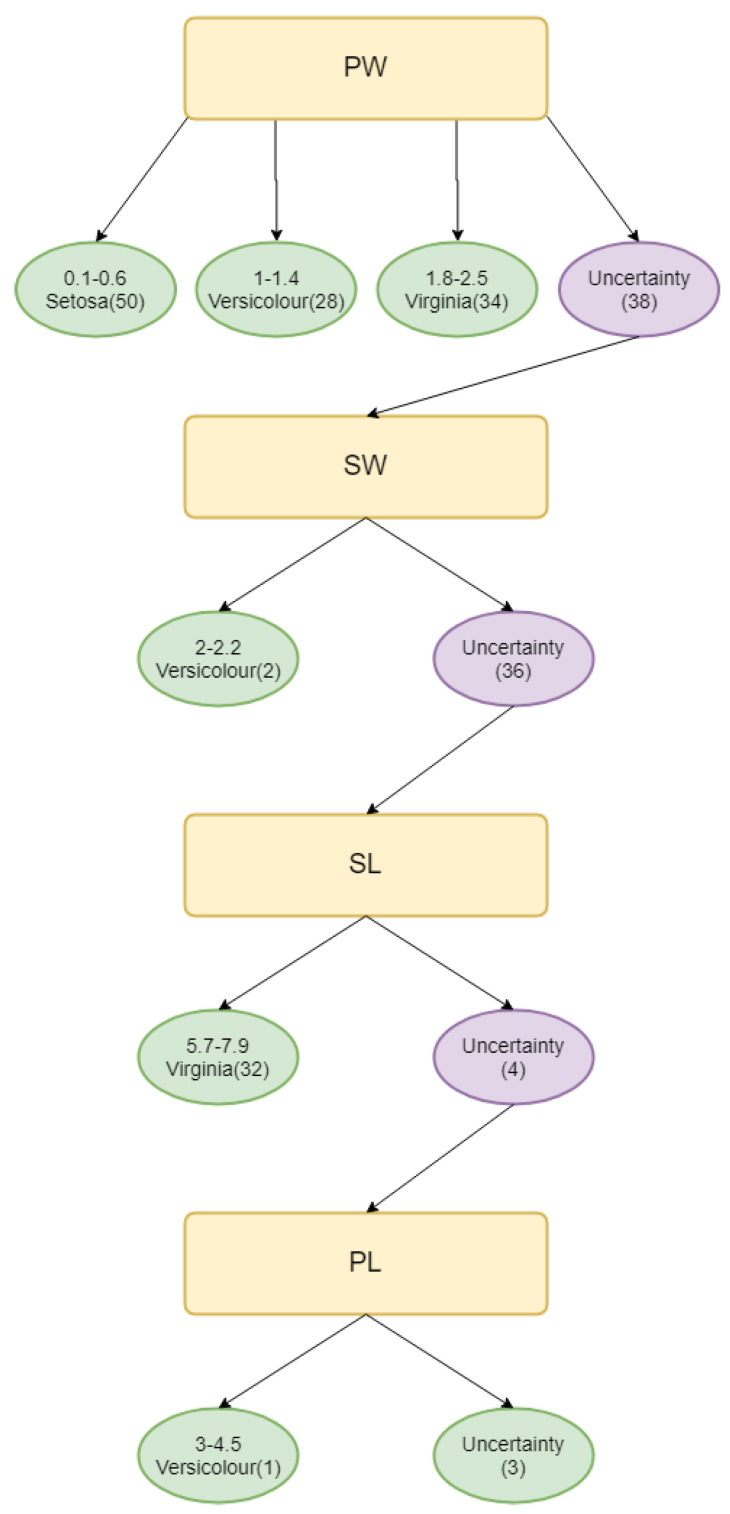
Evidential decision tree for Iris.

**Figure 2:**
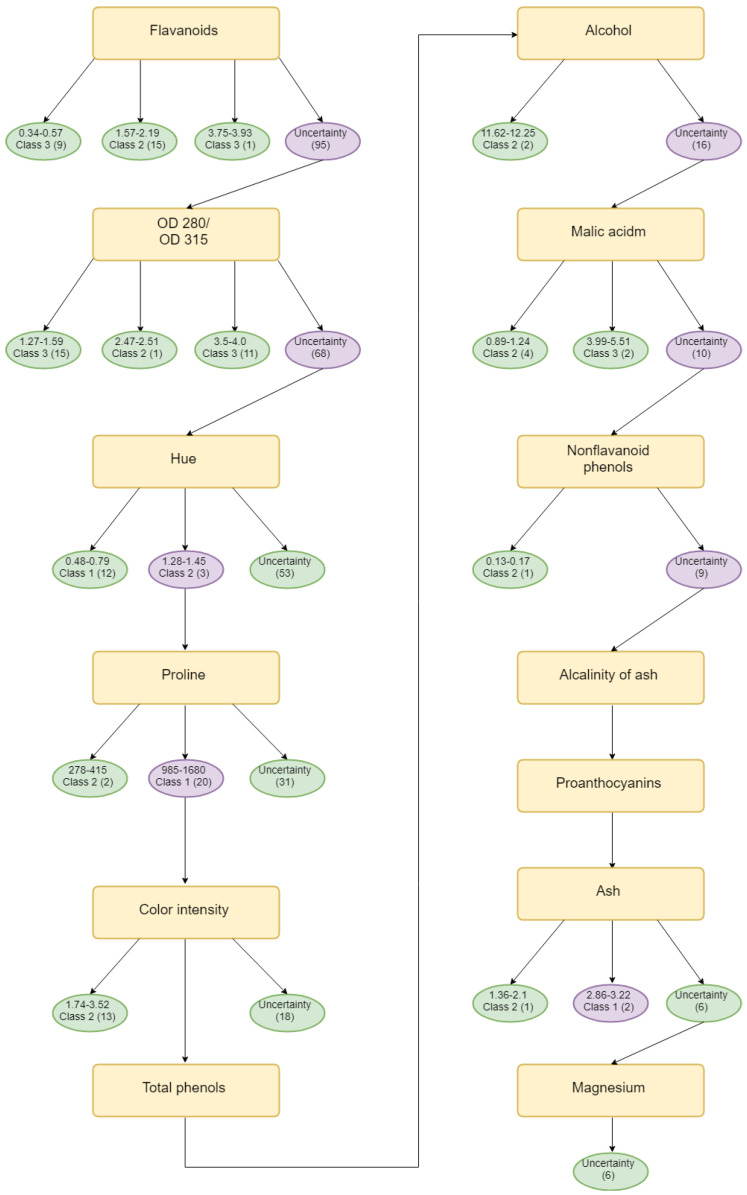
Evidential decision tree for Wine.

Finally, Algorithm 2 is used to find the father nodes of decision tree and Algorithm 3 is used to find the child nodes of decision tree. In the end, the evidential decision tree for iris is constructed and shown in Figure 1; and is shown for wines in Figure 2.

### 4.2. Analysis of the Experiments

**Figure 3:**
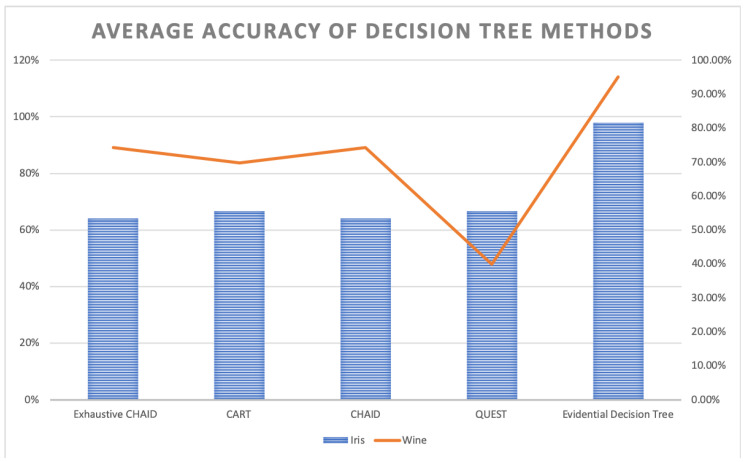
Average Accuracy of Decision Tree Methods.

The evidential decision tree of the **iris** dataset is constructed, as shown in Figure 1. According to SL, SW, PL, and PW’s value of Deng entropy, the father nodes are PW, SW, PL, and SL. Then, according to the designed Algorithm 3, the child nodes are replenished to build the complete decision tree. A total of 150 samples are classified using the entropy decision. As a result, 147 samples can be absolutely classified and 3 samples cannot be classified. Almost 98% of samples are classified under the uncertain situation. In the process of building the evidential decision tree, the lowest value of Deng entropy (PW) firstly is used as the best splitting attribute, which is efficient to classify almost 3/4 of all samples into certain decision attributes. The reason is that the Deng entropy measures the uncertain degree of BBAs. The lower the Deng entropy is, the more accurate the attribute can classify samples. The wine dataset is also used to generate the evidential decision tree for classification. Moreover, there have been more experiments conducted to have a comparison. The average accuracy is 95%, which is much higher than traditional decision methods like Exhaustive CHAID, CART, CHAID, and QUEST. The same applies for wine, shown in Table 7 and Figure 3.

Compared with evidence fusion processing fuzzy data classification, in terms of time complexity of the algorithm, the evidential decision tree is almost O(n) during the process of building the decision tree, which can still complete the task of fuzzy data classification. Instead, the traditional evidence combination methods at least cause $O(n^{2})$ since the orthogonal-sum calculator (Equation (2)) is used in the evidence combination equation. The reason why time complexity of the algorithm increases is that the measurement of Deng entropy is directly used as the indicator of information gain before building the tree.

## 5. Conclusions

The existing methods have been based on Pearson’s correlation coefficient and information entropy to find the best splitting attribute in the process of building a decision tree. However, they are all impossible to handle with uncertain data classification, since Pearson’s correlation coefficient and the traditional information entropy both can only be used in the probability problem. When it comes to uncertain issues, the definition of BBA in D–S evidence theory can be seen as the probability in uncertain problems. Moreover, motivated by the idea of Deng entropy—which can measure the uncertain degree of BBAs—the evidential decision tree is proposed in this paper. The Deng entropy values of attributes’ BBAs are used as the measurement of the best splitting attribute. The lower the Deng entropy is, the more accurate the attribute can classify samples. Without using BBAs combination rules, 98% samples of iris and 95% samples of wine can be classified into certain decision attributes. In other words, the application of the evidential decision tree based on belief entropy efficiently reduces the complexity of algorithms for fuzzy data classification.

## Figures and Tables

**Table 1:** Two Basic Belief Assignments (BBAs) of numerical examples.

Weather	m(A)	m(A,B)	m(A,B,C)	Humidity	m(A)	m(A,C)	m(A,B,C)
$e_{1}$	**0.8**	**0.2**	**0**	$e_{1}$	0.3	0.3	0.4
$e_{2}$	0	0.5	0.5	$e_{2}$	0.2	0.3	0.5
$e_{3}$	0.8	0	0.2	$e_{3}$	**0.4**	**0**	**0.6**
$e_{4}$	0.15	0.15	0.7	$e_{4}$	0	0.8	0.2
$e_{5}$	0.2	0.3	0.5	$e_{5}$	0.8	0	0.2
⋯	⋯	⋯	⋯	⋯	⋯	⋯	⋯

**Table 2:** BBAs of Iris Dataset.

	m(B)	m(B,C)	m(A,B,C)
1	0.6601	0.3246	0.0153
2	0.6772	0.3096	0.0132
3	0.6341	0.3659	0
4	0.5823	0.4177	0
5	0.5407	0.4586	0
6	0.5564	0.4436	0
7	0.8978	0.1022	0
⋯	⋯	⋯	⋯

**Table 3:** Samples Preclassification for Iris.

	SL	SW	PL	PW
Setosa	4.3–5.8	2.3–4.4	1–1.9	0.1–0.6
Versicolour	4.9–5.7	2–3.4	3–5.1	1–1.8
Virginica	4.9–7.9	2.2–3.8	4.5–6.9	1.4–2.5

**Table 4:** Samples Preclassification for Wine.

	**Alcohol**	**Malic acidm**	**Ash**	**Alcalinity of ash**	**Magnesium**
**Class 1**	14.34–14.83		2.86–3.22		
**Class 2**	11.62–12.25	0.89–1.24	1.36–2.1	10.6–11.2/27–30	7.0–8.0/132–162
**Class 3**		3.99–5.51			
	**Total phenols**	**Flavanoids**	**Nonflavanoid phenols**	**Proanthocyanins**	**Color intensity**
**Class 1**	3.52–3.85				
**Class 2**		0.34–0.57/3.75–3.93	0.13–0.17	0.41–0.55/2.96–3.28	1.74–3.52
**Class 3**	0.98–1.1	1.57–2.19			8.7–13.0
	**Hue**	**OD280/OD315**	**Proline**		
**Class 1**	0.48–0.79		985–1680		
**Class 2**	1.28–1.45	2.47–2.51	278–415		
**Class 3**		1.27–1.59/3.5–4.0			

**Table 5:** Deng Entropy for each attribute of Iris.

	SL	SW	PL	PW
Deng Entropy	0.9600	0.8828	0.9865	0.8157

**Table 6:** Deng Entropy for each attribute of Wine.

	**Alcohol**	**Malic acidm**	**Ash**	**Alcalinity of ash**	**Magnesium**
**Deng_Entropy**	196.8	216.2	297.05	268.91	301.28
	**Total phenols**	**Flavanoids**	**Nonflavanoid phenols**	**Proanthocyanins**	**Color intensity**
**Deng_Entropy**	193.08	115.13	263.15	269.62	188.89
	**Hue**	**OD280/OD315**	**Proline**		
**Deng_Entropy**	155.22	148.52	169.15		

**Table 7:** Comparison between traditional decision tree and evidential decision tree.

Average Accuracy	Iris	Wine
**Exhaustive CHAID**	64%	74.20%
**CART**	66.70%	69.70%
**CHAID**	64%	74.20%
**QUEST**	66.70%	39.90%
**Evidential Decision Tree**	98%	95%
